# Understanding Patients’ Adherence-Related Beliefs about Medicines Prescribed for Long-Term Conditions: A Meta-Analytic Review of the Necessity-Concerns Framework

**DOI:** 10.1371/journal.pone.0080633

**Published:** 2013-12-02

**Authors:** Rob Horne, Sarah C. E. Chapman, Rhian Parham, Nick Freemantle, Alastair Forbes, Vanessa Cooper

**Affiliations:** 1 Centre for Behavioural Medicine, Department of Practice and Policy, UCL School of Pharmacy, London, United Kingdom; 2 Department of Primary Care and Population Health, University College London, London, United Kingdom; 3 Department of Internal Medicine, University College Hospital, London, United Kingdom; University of Rochester, United States of America

## Abstract

**Background:**

Patients’ beliefs about treatment influence treatment engagement and adherence. The Necessity-Concerns Framework postulates that adherence is influenced by implicit judgements of personal need for the treatment (necessity beliefs) and concerns about the potential adverse consequences of taking it.

**Objective:**

To assess the utility of the NCF in explaining nonadherence to prescribed medicines.

**Data sources:**

We searched EMBASE, Medline, PsycInfo, CDSR/DARE/CCT and CINAHL from January 1999 to April 2013 and handsearched reference sections from relevant articles.

**Study eligibility criteria:**

Studies using the Beliefs about Medicines Questionnaire (BMQ) to examine perceptions of personal necessity for medication and concerns about potential adverse effects, in relation to a measure of adherence to medication.

**Participants:**

Patients with long-term conditions.

**Study appraisal and synthesis methods:**

Systematic review and meta-analysis of methodological quality was assessed by two independent reviewers. We pooled odds ratios for adherence using random effects models.

**Results:**

We identified 3777 studies, of which 94 (N = 25,072) fulfilled the inclusion criteria. Across studies, higher adherence was associated with stronger perceptions of necessity of treatment, OR = 1.742, 95% CI [1.569, 1.934], *p*<0.0001, and fewer Concerns about treatment, *OR* = 0.504, 95% CI: [0.450, 0.564], *p*<0.0001. These relationships remained significant when data were stratified by study size, the country in which the research was conducted and the type of adherence measure used.

**Limitations:**

Few prospective longitudinal studies using objective adherence measures were identified.

**Conclusions:**

The Necessity-Concerns Framework is a useful conceptual model for understanding patients’ perspectives on prescribed medicines. Taking account of patients’ necessity beliefs and concerns could enhance the quality of prescribing by helping clinicians to engage patients in treatment decisions and support optimal adherence to appropriate prescriptions.

## Introduction

Prescribing medicines is fundamental to the medical management of most long-term conditions. However, approximately half of this medication is not taken as directed, representing a failure to translate potentially effective treatment into optimal outcomes for patients and society [1], [2]. Where prescriptions are appropriate, this level of nonadherence has potentially serious consequences, both for individual patients, in terms of lost opportunities for health gain with increased morbidity and mortality [3], and for the health care system, in terms of wasted resources, increased use of services and hospital admissions [4].

In the absence of a single definitive intervention to address nonadherence [5], the NICE Medicines Adherence Guidelines amalgamate insights from trials of interventions and explanatory studies of nonadherence [1]. They apply a perceptions and practicalities approach [4] recognising that nonadherence may be both unintentional and intentional. Unintentional nonadherence occurs when the patient wants to adhere but is unable to because they lack capacity or resources. For example, they may not have understood the instructions, cannot afford copayment costs, or find it difficult to schedule, administer or remember the treatment. Intentional nonadherence occurs when the patient decides not to follow the recommendations. It is best understood in terms of the perceptual factors (e.g. beliefs and preferences) influencing motivation to start and continue with treatment.

Prescribing consultations do not occur in a vacuum. Patients (and prescribers) bring pre-existing beliefs about the illness and treatment [6], [7] which influence the patient’s evaluation of the prescription, their adherence and even beneficial [8] or adverse outcomes [9]. Interventions to optimise adherence tend to be more effective if they are tailored to the needs of the individual taking account of the perceptions of the treatment as well as practical abilities and resources that enable or impede their adherence [10]. Although the perceptual and practical dimensions of adherence are influenced by the social, cultural, economic and healthcare system contexts, taking account of the patient’s beliefs about the prescribed medication is fundamental to shared-decision making and supporting adherence [1], [11].

Research conducted with patients with a variety of long-term conditions suggests that the key beliefs influencing patients’ common-sense evaluations of prescribed medicines can be grouped under two categories: perceptions of personal need for treatment (Necessity beliefs) and Concerns about a range of potential adverse consequences [7], [12], [13]. This ‘Necessity-Concerns Framework (NCF)’ potentially offers a convenient model for clinicians to elicit and address key beliefs underpinning patients’ attitudes and decisions about treatment.

Over the past decade, a number of studies have been conducted, using a validated questionnaire, the Beliefs about Medicines Questionnaire [14] to quantify Necessity beliefs and Concerns in order to explore the relationship between these beliefs and adherence. This research spans a range of long-term medical conditions, across different settings and within various cultural groups. Many of the individual studies have demonstrated the utility of the NCF in explaining nonadherence to medication (e.g. [15]–[18]). It is therefore timely that a meta-analysis is performed to consolidate the results from these studies and to examine the explanatory value of the NCF in predicting adherence to medication prescribed for long-term medical conditions. In line with the underlying theory, we hypothesized that adherence in long-term conditions would be associated with stronger perceptions of Necessity for treatment and fewer Concerns about adverse consequences.

## Methods

This review was conducted in line with the MOOSE guidelines for meta-analysis of observational trials [19].

### Literature Search

A computerised literature search was conducted by the investigators on April 22^nd^, 2013 using EMBASE, Medline, PsycInfo, CDSR/DARE/CCT and CINAHL. The search strategy included the following terms:

**BMQ or belief$**

***and***

treatment$ ***or*** medicine$ ***or*** medication$

***and***

adheren$ ***or*** complian$

The search was limited to studies published from the year 1999 onwards (the year in which the BMQ was published). Duplicates were removed.

### Inclusion and Exclusion Criteria

Identified studies were included in the meta-analysis if they met the following criteria:

1. participants were suffering from a long-term condition
2. participants were taking medication
3. participants were adults
4. the article was published in a peer-reviewed journal
5. the Necessity and/or Concerns subscales of the BMQ were used
6. a measure of adherence was employed

There were no restrictions based on language, or on cultural or geographical factors.

Titles and abstracts were screened for relevance, and the full text of relevant articles was obtained. Data from each article was extracted as described below.

### Selection of Results When Multiple Relationships between Beliefs and Adherence Were Reported

Fifteen studies reported multiple associations of beliefs related to different adherence measurements (details reported in Table 1). Where the choice was between adherence measures, the most objective measure was selected for the meta-analysis. Therefore, electronic monitoring of adherence [20] and prescription redemption data [16] were chosen over self-report. Where data was presented for both ‘on demand’ and prophylactic medications, data for the prophylactic medication data were chosen [21], [22], for consistency with medications prescribed for other long-term conditions. In studies where cross-sectional and longitudinal data were both available, longitudinal data was used within the analysis [21], [23]–[26]. Where one group provided cross-sectional data at multiple timepoints, the timepoint with the fewest missing data points was selected [27]. If the choice was between two self report measures of adherence, we used the more commonly used measure. Thus the Morisky Medication Adherence Scale (MMAS) was chosen over the Brief Medication Questionnaire [28] and the ACTG adherence measure was used over the Walsh VAS scale [29]. Where patients within a sample were taking multiple medications and individual associations were provided for each medication [30], [31], the mean association was used within the meta-analysis but individual effect sizes are reported in Table 1 to facilitate comparison. Where data on two samples are reported within the same study [32], [33] we included both associations within the analysis.

**Table 1:** Summary Data for Included Studies.

Author and date	Country	Illness Group	N	% male	Mean age (SD)	Study Design	Adherence measure	BMQ (number of items)	*OR*	*p*
Aakre et al.	USA	Comorbid	44	45%	51.1 (9.3)	Cross-	1) Brief Medication	Necessity (5)	1.467	0.523
(2012) [171]		Serious Mental				sectional	Questionnaire	Concerns (6)	0.977	0.969
		Illness and Type					(Antipsychotic	Necessity (5)	4.151	0.024
		II Diabetes					medication)	Concerns (6)	0.673	0.520
							2) Brief Medication Questionnaire (Hypoglycaemic medication)			
Aflakseir	IRN	Type II	102	22%	40.7 (11.4)	Cross-	MARS 10 item version	Necessity (5)	1.670	0.172
(2012) [172]		Diabetes				sectional	see Barnes et al., 2004	Concerns (5)	0.169	<0.001
Aikens et al.	USA	Depression	82	21%	42.9 (10.63)	Cross-	1) General adherence: 4-	Necessity (5)	2.097	0.075
(2005) [28]						sectional	item MMAS^a^	Concerns (5)	0.247	0.001
							2) Recent adherence: 3-	Necessity (5)	3.129	0.008
							item Brief Medication Questionnaire	Concerns (5)	0.333	0.009
Aikens & Piette	USA	Diabetes	803	38%	55.3 (11.8)	Cross-	Single item	Necessity (5)	1.430	0.069
(2009) [173]						sectional		Concerns (6)	0.357	<0.001
Aikens &	USA	Depression	163	38%	35 (10)	Prospective	Brief Medication	Necessity (5)	2.582	0.002
Klinkman (2012) [174]							Questionnaire AND STAR*D Medication Adherence Questionnaire	Concerns (5)	0.683	0.195
Allen LaPointe	USA	Acute Coronary	972	6	Medians for 6	Prospective	Self-report of no	Necessity (5)	1.262	0.137
et al. (2011) [31]		Syndrome		groups	groups		discontinuation nor	Concerns (5)	0.549	<0.001
				in range	between 56-		missed doses in last	Necessity (5)	1.315	0.059
				66–74%	61 SD not		month for 1) ACEI/ARB;	Concerns (5)	0.546	<0.001
					reported		2) Beta-blocker and 3)	Necessity (5)	1.033	0.826
							Lipid-lowering therapy	Concerns (5)	0.488	<0.001
Barnes et al.	NZ	Diabetes	82	Not	European 59.6	Cross-	MARS plus two items re	Necessity (5)	4.054	0.001
(2004) [175]				reported	(12.7); Tongan 59.2 (11.2)	sectional	natural remedies	Concerns (5)	1.670	0.213
										
Batchelder et al.	USA	Comorbid HIV	62	45%	52.8 (7.3)	Cross-	5-item MARS 1)	Necessity	1.300	0.306
(2013) [30]		and Type II				sectional	Antiretroviral 2) Diabetes	Concerns	0.200	0.001
		Diabetes					medication	Necessity	1.050	0.878
								Concerns Unspecified	0.450	0.041
Beck et al.	SWZ	Schizophrenia	150	65.3%	44.9 (11.7)	Cross-	Medication adherence	Necessity (5)	1.942	0.029
(2011) [176]		or Schizoaffective Disorder				sectional	subscale of the Service Engagement Scale (Tait et al., 2002)- clinician rated. Brief Adherence Rating Scale (BARS; Byerly et al., 2008) BARS selected for use here	Concerns (5)	0.775	0.396
Berglund et al.	SWE	Statin Users	414	50.8%	64.2 (9.5)	Cross-	4-item MMAS	Necessity (5)	2.266	<0.001
(2013) [177]						sectional		Concerns (5)	1.338	0.105
Bhattacharya et	UK	Colorectal or	43	44.2%	64.5 (7.4)	Cross-	5-item MARS	Necessity (5)	1.408	0.562
al. (2012) [178]		Breast Cancer				sectional		Concerns (5)	0.570	0.352
Brown et al.	USA	Depression	192	29%	45.2 (16.0)	Cross-	4-item MMAS	Necessity (5)	1.235	0.425
(2005) [179]						sectional (Longitudinal study but only baseline results reported)		Concerns (5)	0.362	<0.001
Brown et al. (2013) [160]	USA	HIV	116	58%	45.3 (8.6)	Cross-sectional	VAS scale 0–100% used to rate adherence to each medication over the last month dichotomized at 95%	Necessity (8)	2.357	0.014
Butler et al.	UK	Renal	58	66%	48.0 (13)	Cross-	Electronic monitors^b^	Necessity (5)	4.871	0.003
(2004) [180]		Transplant				sectional		Concerns (7)	0.517	0.184
Byer & Myers	UK	Asthma	64	50%	39.6 (13.83)	Cross-	1) Number of preventer	Necessity (5)	5.915	0.001
(2000) [16]						sectional	inhaler prescriptions	Concerns (5)	–	–
							collected^a^	Necessity (5)	3.129	0.05
							2) Number of reliever	Concerns (5)	–	–
							inhaler prescriptions	Necessity (5)	5.915	0.001
							collected	Concerns (5)	–	–
							3) Self-reported adherence			
Byrne et al.	IRE	Coronary Heart	1084	65%	66.0 (9.1)	Cross-	5-item MARS	Necessity (5)	2.551	<0.001
(2005) [17]		Disease				sectional		Concerns (5)	0.669	<0.001
Chisholm-Burns	USA	Renal	512	61.1%	52.4 (10.7)	Cross-	Immunosuppressant	Necessity (5)	2.065	<0.001
et al.		Transplant				sectional	Therapy Adherence Scale	Concerns (5)		
(2012) [181]							(ITAS) <12 non-adherence			
Clatworthy et al.	UK	Bipolar	223	36%	48 (11.2)	Cross-	5-item MARS	Necessity (5)	2.114	0.006
(2009) [18]		Disorders				sectional		Concerns (6)	0.371	0.001
Clifford et al.	UK	Chronic illness	146	52%	64.3 (12.06)	Longitudinal	Telephone call (“When	Necessity (5)	1.764	0.090
(2008) [142]							was the last time you missed a dose of this medicine?”). Nonadherence defined as any dose missed in the previous 7 days^b^	Concerns (5)	0.457	0.020
Cooper et al.,	UK	HIV	234	84%	42 (8.9)	Longitudinal	At 48 weeks MASRI	Necessity (15)	1.863	0.010
(2011) [182]							(Walsh et al., 2002) scale- VAS % taken over last month dichotomized at 95%	Concerns (8)	0.499	0.004
de Boer-van der	NTL	HIV	341	90%	45	Cross-	Self report % of	Necessity (8)	1.600	0.018
Kolk et al. (2008) [183]						sectional	prescribed medicines taken	Concerns (11)	0.070	0.075
De Las Cuevas	ESP	Affective	167	23.4%	56.1 (12.3)	Cross-	4-item MMAS	Necessity (5)	1.111	0.710
et al. (2013) [184]		Disorders				sectional		Concerns (5)	2.521	0.002
De Smedt et al.	NTL	Heart Failure	960	63.6%	69.6 (11.9)	Cross-	SECope non-adherence	Necessity (5)	1.257	0.616
(2012) [185]						sectional	subscale (Johnson & Neilands, 2007)	Concerns (5)	0.484	0.112
de Thurah et al.	DMK	Rheumatoid	91	36%	Median 63	Prospective	CQ-R 1) 9 months 2)	Necessity (5)	9.600	<0.001
(2010) [21]		Arthritis					baseline	Concerns (5)	0.420	0.132
								Necessity (5)	3.630	0.016
								Concerns (5)	0.793	0.652
Ediger et al	CAN	IBD	326	40%	41.0 (14.06)	Cross-	5-item MARS^b^	Necessity (5)	1.522	0.039
(2007) [186]						sectional		Concerns (5)	0.677	0.054
Emilsson et al.	SWE	Asthma	35	28.6%	52.9 (14.7)	Cross-	Pill count	Necessity (5)	4.438	0.032
(2011) [187]						sectional		Concerns (5)	0.555	0.365
Fawzi et al.	EGT	Depression or	108	33.3%	61.3 (5.3)	Cross-	10-item MARS	Necessity (5)	3.712	0.001
(2012) [188]		Adjustment Disorder with Depressed Mood				sectional	(Thompson et al., 2000) MARS chosen and GAM (global adherence measure- 1 item)	Concerns (5)	0.269	0.001
Foo et al.	SGP	Glaucoma	344	64.8%	66.1 (10.2)	Cross-	8-item MMAS dichot. at	Necessity (4)	1.045	0.837
(2012) [189]						sectional	8	Concerns (5)	2.778	<0.001
French et al.	UK	Type II	453	57.4%	65.9 (10)	Prospective	5-item MARS 1) Baseline	Necessity (5)	1.295	0.232
(2013) [23]		Diabetes					2) Prospective	Concerns (5)	0.525	0.004
								Necessity (5)	1.800	0.013
								Concerns (5)	0.116	<0.001
Gauchet et al.	FRA	HIV	127	78%	39.7 (9.2)	Cross-	16-item self-report	Necessity (5)	3.264	0.001
(2007) [190]						sectional	measure (devised by authors)	Concerns (5)	0.865	0.656
Gatti et al.	USA	Chronic illness	275	27%	-	Cross-	8-item MMAS dichot. at	Necessity (5)	1.239	0.331
(2009) [191]						sectional	1	Concerns (6)	0.357	<0.001
George &	CAN	Heart Failure	350	69%	61.0 (12.6)	Cross-	1) Prescription dispensing	Necessity (5)		
Shalansky						sectional	data (nonadherence	Concerns (5)	1.529	0.069
(2007) [192]							defined as <90% mean refill adherence)^b^2) 4-item MMAS^c^		0.954	0.839
Gonzalez et al.	USA	HIV	325	60%	40.9 (8.5)	Longitudinal	1) ACTG	Necessity (8)	1.494	0.048
(2007) [20]						randomised	2) MEMS cap – one drug	Concerns (11)	0.459	<0.001
						trial	in each participant’s	Necessity (8)	1.494	0.048
							regimen monitored, usually the protease inhibitor (% adherence)^a^	Concerns (11)	0.720	0.106
Griva et al.	UK	Kidney	218	59.6%	49.7 (12.3)	Cross-	5-item MARS item plus	Necessity (5)	7.278	<0.001
(2012) [193]		Transplant				sectional	serum immunosuppressant concentrations	Concerns (5)		
Grunfeld et al	UK	Breast Cancer	110	0%	56.3 (7.0)	Cross-	1) Asked “In the past^c^	Necessity (5)	2.916	0.007
(2005) [194]						sectional	week have you taken your tamoxifen everyday?” (Yes/No)^b^ 2) 5-item MARS	Concerns (5)	0.868	0.708
Hedenrud et al.	SWE	Migraine	174	16%	Not calculable	Cross-	5-item MARS^b^	Necessity (5)	0.747	0.309
(2008) [195]						sectional		Concerns (5)	0.588	0.064
Horne et al.	UK	Cardiac and	210	49%	50.8 (16.2)	Cross-	4-item RAM	Necessity (5)	2.018	0.006
(1999) [14]		General Medical (pooled data)				sectional		Concerns (5)	0.347	<0.001
Horne &	UK	Asthma, Renal	324	54%	54.1 (15.96)	Cross-	4-item MARS	Necessity (5)	2.180	<0.001
Weinman (1999) [7]		Cardiac, Oncology (pooled data)				sectional		Concerns (5)	0.281	<0.001
Horne et al.	UK	Renal	47	49%	49.0 (17.3)	Cross-	Single item: ‘How often	Necessity (5)	1.115	0.842
(2001) [196]		(Haemodialysis)				sectional	do you deliberately miss a dose of medication?’	Concerns (5)	0.215	0.010
Horne &	UK	Asthma	100	39%	49.3 (18.1)	Cross-	9-item MARS	Necessity (6)	3.405	0.002
Weinman						sectional		Concerns (11)	0.178	<0.001
(2002) [166]										
Horne et al.	UK	HIV	109	97%	41.2 (9.0)	Cross-	Single item: ‘How much	Necessity (8)	1.773	0.126
(2004) [197]						sectional	of your HAART medication did you take within two hours of when you were supposed to?’^b^	Concerns (11)	0.524	0.095
Horne et al.	UK	HIV	117	96%	37.8 (8.4)	Prospective	Single item: VAS from	Necessity (6)	2.477	0.008
(2007) [198]						follow-up	MASRI^b^	Concerns (7)	0.298	<0.001
Horne et al.	UK	IBD	1871	37%	50 (16.0)	Cross-	4-item MARS	Necessity (8)	1.790	<0.001
(2009) [167]						sectional		Concerns (9)	0.600	<0.001
Horne et al.	UK	Hypertension	230	88%	67.6	Prospective	1) 6- item MARS–	Necessity (5)	1.675	0.096
(2010) [24]							baseline	Concerns (6)	0.464	0.013
							2) 6-item MARS	Necessity (5)	1.007	0.987
							Prospective (Compared to tablet count for 48% of sample)	Concerns (6)	0.195	<0.001
Hou et al.	UK	Bipolar	35	28.6%	45 (11)	Cross-	MMAS 4-item (dichot. at	Necessity (5)	0.881	0.837
(2010) [199]		Affective Disorder				sectional	1)	Concerns (5)	0.680	0.532
Hunot et al.	UK	Depression	178	25%	40.1 (12.6)	Longitudinal	1) Single item: current	Necessity (5)	3.346	<0.001
(2007) [200]							antidepressant use/non-use (“Are you currently taking antidepressants?”)^b^ 2) MARS^c^ 3) Prescription refill data^c^	Concerns (6)	0.223	<0.001
Iihara et al	JPN	Hospital	151	62.3%	–	Cross-	Measure based on MMAS	Necessity (5)	1.998	0.020
(2010) [201]		Inpatients				sectional		Concerns (5)	0.593	0.079
Johnson et al.	USA	HIV	295	100%	45.2 (10.1)	Cross-	1) ACTG 3 days (%	Necessity (5)	0.960	0.365
(2012) [29]						sectional	taken) dichot. at 100%^a^	Concerns (5)	0.930	0.058
							2) Walsh VAS 0–100%	Necessity (5)	1.020	0.572
							last 30 days dichot at 100%	Concerns (5)	0.960	0.273
Jonsdottir et al.	UK	Schizophrenia/	280	51%	35.1	Cross-	VAS (0%–100%)	Necessity (8)	5.887	<0.001
(2009) [202]		Bipolar disorder				sectional		Concerns (9)	0.493	0.057
Kemp et al.	UK	Epilepsy	37	51%	40.7 (SD not	Cross-	Low-dose of	Necessity (5)	0.441	0.200
(2007) [203]					reported)	sectional	phenobarbital indicative of nonadherence, and/or measurement of antiepileptic drug levels	Concerns (5)	0.599	0.414
Khanderia et al.	USA	Coronary Artery	132	83%	65.8 (10.1)	Cross-	4-item MMAS^b^	Necessity (5)	1.050	0.875
(2008) [204]		Bypass Graft				sectional		Concerns (5)	0.584	0.092
Kressin et al.	USA	Hypertension	806	35%	59	Cross-	Hill-Bone Compliance to	Necessity (5)	1.414	0.200
(2010) [205]						sectional	High Blood Pressure Therapy Scale, 9 item adherence subscale	Concerns (5)	0.525	<0.001
Kronish et al	USA	Stroke or TIA	600	60.6%	63.4 (11.2)	Cross-	8-item MMAS dichot. at	Necessity (5)	1.120	0.557
(2013) [206]						sectional	> = 6	Concerns (4) (modified items)	0.193	<0.001
Kung et al.	NZ	Heart, Liver,	326	64.4%	Heart	Cross-	Immunosuppressant	Necessity (5)	1.605	0.021
(2012) [207]		Lung Transplant			transplant: 54.4 (11.8) Lung transplant 49.3 (13.1) Liver transplant 55.1 (12.3)	sectional	Therapy Adherence Scale (ITAS) <12 non-adherence	Concerns (5)	0.493	0.001
Llewellyn	UK	Haemophilia	65	100%	36.4 (12.2)	Cross-	1) Adherence to	Necessity (5)	5.915	0.001
et al. (2003) [22]						sectional	frequency of prophylactic	Concerns (5)	0.599	0.270
							infusion with clotting	Necessity (5)	4.241	0.004
							factor^a^ 2) Adherence to recommended ‘on demand’ dose of clotting factor 3) Adherence to recommended dose of clotting factor^c^	Concerns (5)	0.897	0.813
Maguire et al.	UK	Hypertension	327	46%	Not reported	Cross-	4-item RAM	Necessity (5)	0.665	0.242
(2008) [208]						sectional		Concerns (5)	0.422	0.014
Mahler et al.	GMY	Mixed Chronic	360	53.3%	69.5 range 19–	Cross-	5-item MARS D	Necessity (5)	2.097	<0.001
(2012) [209]		Illness			95	sectional		Concerns (5)	0.515	0.001
Maidment	UK	Depression	67	49%	74.2 (6.1)	Cross-	Global Adherence	Necessity (5)	3.002	0.020
et al. (2002) [15]		(older adults)				sectional	Measure (single rating by interviewer)	Concerns (5)	0.247	0.004
Menckeberg et	NTL	Asthma	238	33%	36.2 (6.3)	Cross-	5-item MARS	Necessity (9)	3.878	<0.001
al. (2008) [210]						sectional		Concerns (12)	0.496	0.004
Moshkovska et	UK	Ulcerative	169	51%	49 (SD not	Cross-	1) 12 item study specific	Necessity (5)	1.976	0.002
al. (2009) [211]		Colitis			reported)	sectional	self report questionnaire	Concerns (6)	0.639	0.035
Nakhutina et al.	USA	Epilepsy	72	37.5%	44 (14.2)	Cross-	4-item MMAS	Necessity (5)	1.388	0.455
(2011) [212]						sectional		Concerns (5)	0.694	0.406
Neame &	UK	Rheumatoid	344	33%	49.5% aged	Cross-	Single item: ‘I often do	Necessity (5)	0.885	0.737
Hammond (2005) [213]		Arthritis			over 65	sectional	not take my medicines as directed’^b^	Concerns (5)	0.313	0.002
Nicklas et al.	UK	Chronic Pain	217	–	–	Cross-	6-item MARS	Necessity (5)	2.018	0.005
(2010) [214]						sectional		Concerns (5)	0.645	0.079
O’Carroll et al.	UK	Liver	33	52%	55.8 (13.37)	Cross-	1) ‘Medication adherence’	Necessity (5)	1.734	0.411
(2006) [215]		Transplant				sectional	factor of the Transplant Effects Questionnaire (TxEQ) 2) 5-item MARS^c^	Concerns (5)	0.137	0.009
O’Carroll et al.	UK	Ischaemic	180	54%	69 (11.4)	Cross-	5-item MARS with	Necessity (5)	0.705	0.202
(2011) [25]		Stroke				sectional	salicyclic acid/creatinine	Concerns (5)	0.209	<0.001
							1) Baseline	Necessity (5)	0.778	0.359
							2) Prospective	Concerns (5)	0.328	<0.001
Ovchinikova et	AUS	Asthma	134	31%	53 (19)	Longitudinal	MARS 1) Baseline 2)	Necessity (5)	1.429	0.262
al. (2011) [26]							Prospective	Concerns (5)	0.220	<0.001
								Necessity (5)	1.328	0.387
								Concerns (5)	0.278	<0.001
Percival et	AUS	Heart Failure	43	83.7%	64.2 (17.1)	Cross-	5-item MARS dichot. at	Necessity (5)	3.068	0.165
al.(2012) [216]						sectional	23	Concerns (5)	0.508	0.399
Peters et al.	USA	Marfan	174	42%	39.8 (12.2)	Cross-	3-item self-report measure	Necessity (5)	1.299	0.417
(2001) [217]		Syndrome				sectional	(adapted from MARS)	Concerns (5)	0.424	0.010
Phatak &	USA	Hypertension,	250	38%	<30 (11.2%)	Cross-	9-item MMAS	Necessity (5)	1.550	0.059
Thomas		Arthritis, Back			30–39 (14%)	sectional		Concerns (6)	0.215	<0.001
(2006) [218]		Problems,			40–49 (37.2%)					
		Asthma,			50–59 (24.4%)					
		Hypercholesterolemia			>60 (13.2%)					
Rajpura &	USA	Hypertension	117	64.1%	55–65 (23.9%)	Cross-	MMAS	Necessity (5)	2.551	0.008
Nayak (2013)		and aged 55 or over			>65 (52.1%)	sectional		Concerns (5)	0.423	0.014
Rees et al.	AUS	Glaucoma	131	61.1%	67.7 (13.6)	Cross-	4-item RAM	Necessity (5)	1.966	0.035
(2010) [219]						sectional		Concerns (8)	0.651	0.180
Rees et al.	USA,	Glaucoma	475	55.4%	African	Cross-	4-item RAM	Necessity (5)	2.385	<0.001
(2013) [220]	SGP, AUS				Americans: 69.6 (12.4) White Americans: 68.65 (13.0) Australians: 69.2 (13.1) Singaporeans: 65.1 (11.8)	sectional		Concerns (8)	0.414	<0.001
Reynolds et al	USA	Osteoporosis	193	0%		Cross-	Osteoporosis Specific 8-	Necessity (5)	3.405	<0.001
(2012) [221]						sectional	item MMAS	Concerns (6)	0.424	0.005
Ross et al.	UK	Hypertension	515	52%	59.9 (12.16)	Cross-	4-item MMAS^b^	Necessity (5)	3.060	0.001
(2004) [159]						sectional		Concerns (5)		
Ruppar et al.		Hypertension	33	21%	70.6 (9.1)	Prospective	MEMS for 6 weeks post-	Necessity (5)	0.501	0.306
(2012) [222]							BMQ	Concerns (5)	0.254	0.053
Russell &	NZ	Depression	85	28%	43.7 (11.5)	Cross-	5-item MARS	Necessity (5)	1.115	0.786
Kazantzis (2008) [223]						sectional		Concerns (14)	0.269	0.002
Schoenthaler et	USA	Type II	608	48%	62.1 (9.2)	Cross-	MPR over last 2 years	Necessity (5)	0.757	0.060
al. (2012) [224]		Diabetes				sectional		Concerns (5)	0.878	0.380
Schuz et al.	GMY	Older Adults	309	59.3%	73.3 (5.1)	Longitudinal	2 items from RAM	Necessity (2)	1,353	0.155
(2011) [225]		with Comorbid Illnesses						Concerns (2)	0.590	0.014
Shiyanbola &	USA	Diabetes	16	0%	46.1 (10.2)	Cross-	4-item MMAS	Necessity (5)	0.917	0.931
Nelson (2011) [226]						sectional		Concerns (5)	1.539	0.671
Sirey et al.	USA	Older Adults	299	22.1%	Nonadherent	Cross-	4-item MMAS	Necessity (5)	1.182	0.435
(2013) [227]		with Comorbid Illnesses			75.6 (7.3); Adherent 76.7 (7.4)	sectional		Concerns (5)	0.494	0.001
Sofianou et al.	USA	Asthma	242	16.1%	67.4 (6.8)	Cross-	10-item MARS	Necessity (5)	2.353	<0.001
(2012) [228]						sectional		Concerns (5)	0.437	0.001
Tibaldi et al.,	Italy	Chronic illness	427	45%	59 (14)	Cross-	5-item MARS	Necessity (5)	1.314	0.123
(2009) [229]						sectional		Concerns (6)	0.488	<0.001
Sud et al.,	USA	Acute Coronary	208	60.6%	64.9 (13.0)	Cross-	4-item MMAS	Necessity (5)	1.800	0.022
(2005) [60]		Syndrome				sectional		Concerns (5)	0.720	0.198
Trachtenberg et	USA, UK	Thalassemia	371	47.4%	24.0 (12.6)	Longitudinal	Self-reported number of	Necessity (5)	0.694	0.256
al. (2012) [32]							doses taken in the past	Concerns (5)	0.964	0.910
							week and month 1) DFO	Necessity (5)	1.115	0.633
							2) Oral iron chelator; serum ferritin, liver biopsy, liver iron concentration.	Concerns (5)	0.720	0.152
Treharne et al.	UK	Rheumatoid	85	25%	58.9 (12.64)	Cross-	1) 19-item CQR	Necessity (5)	31.758	<0.001
(2004) [230]		Arthritis				sectional	2) 2 items from the MARS^c^	Concerns (5)	0.621	0.239
Unni & Farris	USA	Cholesterol	420	54.4%	Cholesterol:	Cross-	Medication Adherence	Necessity (5)	0.981	0.925
(2011)a [33]		Loweing			59.4; Asthma:	sectional	Reasons Scale (4 types of	Concerns (5)	0.265	<0.001
		Medication or			48.7		non-adherence for each	Necessity (5)	1.714	0.004
		Asthma Maintenance Medication Patients					medication combined into any or none)	Concerns (5)	0.506	<0.001
Unni & Farris	USA	Older Adults	1061	45.6%	Adherent:	Cross-	4-item MMAS 1) time 1;	Necessity (5)	1.010	0.931
(2011)b [27]					73.2 (9.2)	sectional	2) time 2	Concerns (5)	0.462	<0.001
					Non-adherent:	(two time		Necessity (5)	1.075	0.560
					72.5 (5.5)	points)		Concerns (5)	0.503	<0.001
Uusküla et al.	EST	HIV	161	55%	≤30 N = 45	Cross-	Recall of proportion of	Necessity (6)	1.516	0.442
(2012) [231]					>30 N = 82	sectional	total doses prescribed taken during past 3 days	Concerns (7)	0.250	0.073
Van den Bemt	NTL	Rheumatoid	228	33%	56.2 (12.2)	Cross-	Self-report	Necessity (5)	1.516	0.442
et al. (2009) [232], [233]		Arthritis				sectional		Concerns (5)	0.392	<0.001
Voils et al.	USA	Hypertension	201	86%	64.1 (11.0)	Cross-	8-item MMAS	Necessity (5)	1.516	0.442
(2012) [233]						sectional		Concerns (5)	0.392	<0.001
Wileman et al.	UK	End-Stage	76	60.5%	63.1 (15.4)	Cross-	Medications adherence	Necessity (5)	1.641	0.270
(2011) [234]		Renal Disease				sectional	quesionnaire (MAQ) plus serum phosphate level > = 1.8 mmol/l	Concerns (5)	0.750	0.521
Wong &	UK	Rheumatoid	68	40%	55.8 (13.0)	Longitudinal	Patient report of drug	Necessity (5)	1.319	0.568
Mulherin (2007) [235]		Arthritis					continuation at 1 year versus discontinuation^b^	Concerns (5)	0.870	0.774
Yu et al.	SGP	Peritoneal	20	60%	64.4 (11.6)	Cross-	Specially designed 5 item	Necessity (5)	1.828	0.499
(2012) [236]		Dialysis				sectional	scale with 5 non-adherent behaviours, rated on 5 point Likert scale plus serum phosphate >1.78 mmol/l	Concerns (5)	0.913	0.918
Zerah et al.	FRA	Patients taking	182	21%	Median 47	Cross-	4-item MMAS	Necessity (5)	2.008	0.042
(2012) [237]		Glucocorticoids			[range 33–61]	sectional		Concerns (5)	0.484	0.035

*Note.* NZ = New Zealand; IRE = Ireland; NTL = Netherlands; CAN = Canada; FRA = France; SWE = Sweden; IRN = Iran; SWZ = Switzerland; ESP = Spain; DMK = Denmark; EGT = Egypt; SGP = Singapore; JPN = Japan; EST = Estonia; GMY = Germany; AUS = Australia; IBD = inflammatory bowel disorder; TIA = Transient Ischemic Attack; MARS is the Medication Adherence Rating Scale from Thompson, Kulkarni, & Sergejew (2000); MEMS is Medication Event Monitoring System; CQ-R is the Compliance Questionnaire-Rheumatology from de Klerk, van der Heijde, Landewé, van der Tempel, & van der Linden (2003); MMAS is the Morisky Medication Adherence Scale from Morisky, Green, & Levine (1986); TxEQ is the Transplant Effects Questionnaire from Ziegelmann et al. (2002); ACTG is the Adherence to Combination Therapy Guide from Chesney et al., 2000; RAM is the Reported Adherence to Medication Scale from Horne et al., (1999), renamed MARS (Medication Adherence Report Scale); VAS = visual analogue scale.

a Adherence result selected for use in meta-analysis;

b Adherence measure dichotomised into adherent and nonadherent groups;

c Relationship between adherence measure and BMQ scales not reported.

### Data Extraction

The following information was extracted from papers onto coding forms: author names, date of publication, the country in which the research was conducted (dichotomized into UK or non-UK), sample size, illness group, sex (% male), mean age, study design (cross-sectional, longitudinal or prospective), the number of Necessity and Concerns items included (since items may be added specific to the medication prescribed), the adherence measure used, information (means and standard deviations, odds ratios and 95% confidence intervals or correlation coefficients) to calculate the effect size between adherence and Necessity beliefs and Concerns, and the p-value. Where the full required statistics were not reported, authors were contacted for further information.

### Methodology/Quality Assessment

A simple methodology assessment tool was devised for this study. Methodology was assessed by two of three independent expert raters (SC, RP and VC) using the following parameters:

study location (UK or non-UK)
study design (cross-sectional or longitudinal/prospective)
measure of adherence (self-report or objective measure [electronic monitors, prescription redemption, blood test results]).
sample size (<82 = 0 or ≥82 = 1). This was based on the sample needed to detect a medium effect size for a correlation (*r* = 0.3) with an alpha level of 0.05 and 80% power.

Ratings were completed independently and then combined. There were no disagreements regarding ratings.

### Statistical Analysis

The primary outcome measure was adherence to medication. For each study, the effect size was expressed as an odds ratio with 95% confidence intervals. Where studies reported the standard mean difference or correlation coefficient, the effect size was converted into an odds ratio, using the Comprehensive Meta-Analysis program. We used a random effects model to accommodate heterogeneity between studies which was anticipated due to differences with respect to sample characteristics, study design and the adherence measure used.

The presence of significant heterogeneity across studies was examined using the chi-squared statistic (*Q*). The magnitude of this heterogeneity across studies was estimated using the *I* ^2^ statistic [34], which assesses the percentage of variance among studies which is not due to chance.

Sensitivity analyses were conducted to ascertain whether the effect sizes seen were robust when individual studies, or studies grouped based on the methodological factors described above were excluded.

Orwin’s fail-safe *N* [35], [36] was calculated to estimate the number of unpublished studies necessary to reverse any conclusion that a significant effect exists (based on the conservative assumption that unpublished studies would have effect sizes of equal magnitude but opposite direction to the overall effect size in this meta-analysis). Egger’s t-test and funnel plots were also used to test for publication bias, in line with recent recommendations [37].

## Results

### Selection of Studies

**Figure 1:**
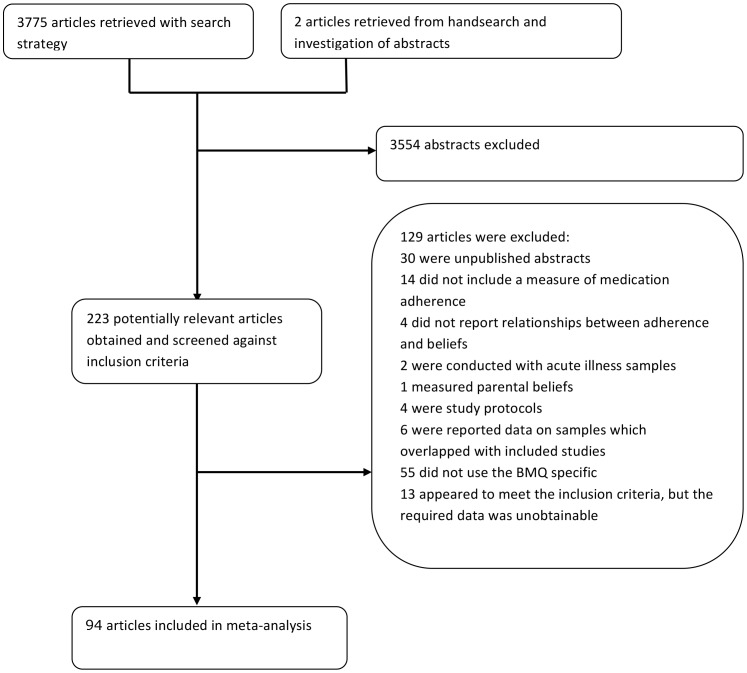
Selection process for study inclusion.

Ninety-four percent (3554) of the 3775 studies retrieved were rejected after checking the titles and abstracts against the selection criteria above (Figure 1). 223 relevant articles were identified. A search of the reference lists of these articles revealed one further relevant study [38].

Of the 223 studies identified, a further 129 were excluded (Figure 1). Thirty of these were unpublished studies and conference proceedings. These were investigated further and authors were contacted where necessary to clarify whether unpublished work had led to publications [39]–[45]. Sixteen studies [44], [46]–[59] [60] had since been published, fifteen of which already formed part of the included list and one additional eligible study was available online early [61]. Six papers reported data on samples which overlapped with included studies [62]–[67], and four were protocols for ongoing studies [68]–[71].

Thirteen studies were excluded because they did not include a measure of medication adherence [72]–[85]. Two of these included separate assessment modes for intentional and unintentional adherence but no overall adherence assessment [80], [85]. Fifty-five studies did not use the BMQ Specific scales [86]–[140]. Four studies were excluded because the relationship between treatment beliefs and adherence behaviour was not reported [24], [141]–[143]. Two articles were conducted in acute rather than long-term condition samples (influenza [144] and antibiotic use [145]) and one article was excluded because *parental* beliefs about medicine were measured [146]. Thirteen studies study met the inclusion criteria but the article did not contain the required statistical information. We contacted the authors but were unable to obtain the relevant data [38], [147]–[158]. Thus, once screened against the inclusion criteria, 94 articles were retained for inclusion in the meta-analysis. Table 1 provides a summary of each of the studies included in the meta-analysis.

Three of the included studies [16], [159], [160] reported associations between adherence and Necessity beliefs, but not Concerns. The authors of these articles were contacted, but the data for Concerns was unavailable. Two studies [32], [33] reported two largely non-overlapping samples for both Necessity beliefs and Concerns. Thus, data for 91 studies and 93 comparisons for Concerns, and data for 94 studies and 96 comparisons for Necessity beliefs, were included in the meta-analysis.

### Sample Characteristics

The mean age of participants in the 94 included studies ranged from 24.0 to 74.2, with an overall mean age of 55.8 (it was not possible to calculate the mean age in 13 studies). The percentage of males ranged from 0–100% (breast cancer and haemophilia samples respectively), with an overall percentage of males of 49.7% male (excluding 3 studies where it was not possible to calculate the number of males). Sample sizes ranged from 16 to 1871.

The total sample, *N* = 25,072, encompassed patients with asthma, renal disease, organ transplantation, dialysis chronic pain, kidney transplantation, cancer, cardiovascular disorders, Marfan’s syndrome, depression, haemophilia, diabetes, HIV, rheumatoid arthritis, osteoporosis, thalassemia, inflammatory bowel disease, bipolar disorder, schizophrenia, epilepsy, migraine, back problems, glaucoma and mixed chronic illness.

Thirty-three studies (35.1%) used the MARS to measure adherence, 20 used the Morisky Medication Adherence Scale (21.2%), 3 used pharmacy refill (3.2%), 3 used electronic monitoring (3.2%) and two or fewer studies used the remaining measures.

### Effect Sizes

#### Necessity beliefs

There was a significant relationship between Necessity beliefs and adherence, *OR* = 1.742, 95% CI [1.569, 1.934], *p*<0.0001. There was significant heterogeneity between the 96 comparisons from 94 studies, *Q*(95) = 422.662, *p*<0.001, which was substantial in magnitude, *I* ^2^ = 77.52%.

**Figure 2:**
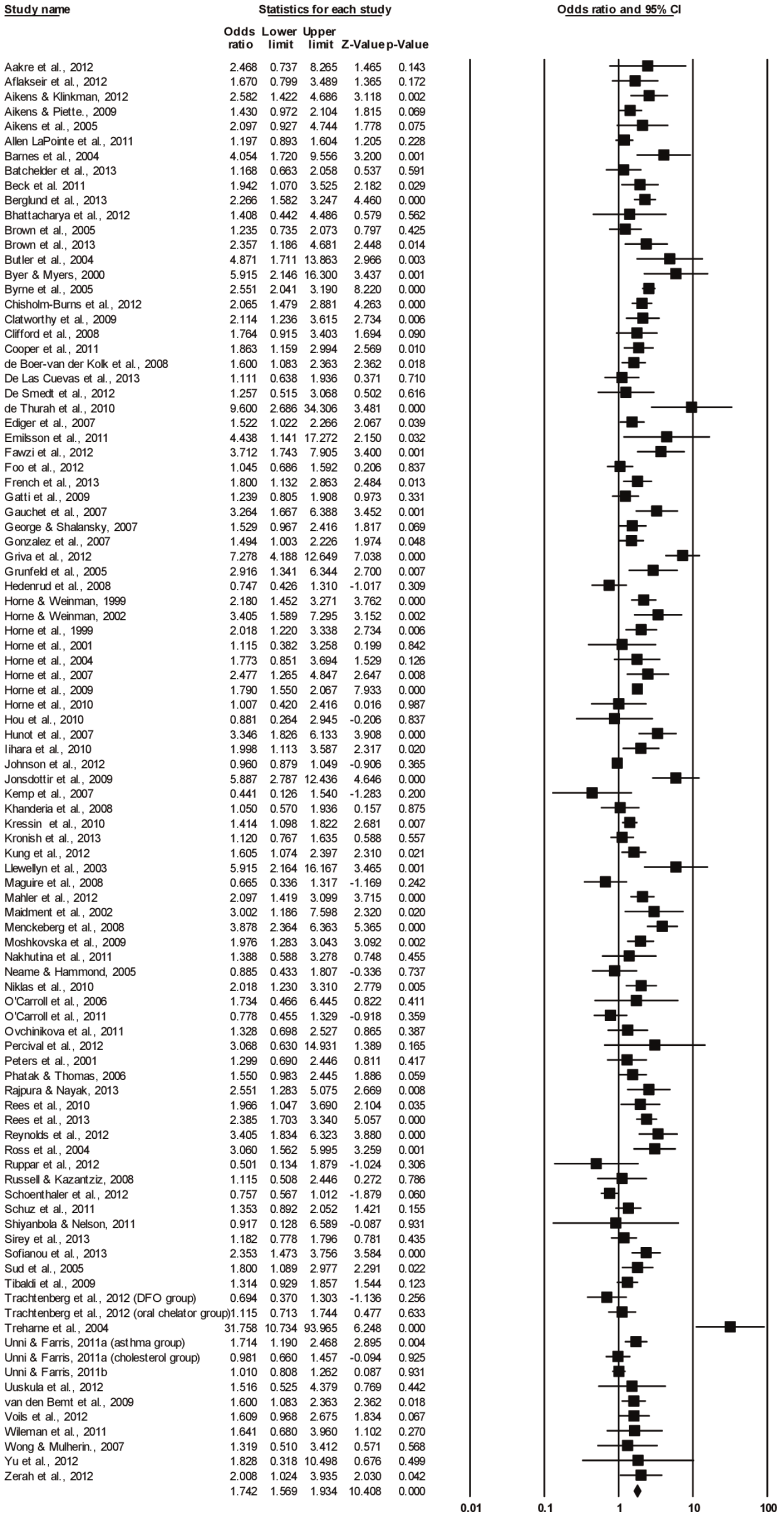
Forest plot of effect sizes for BMQ Necessity and medication adherence.

Figure 2 presents the individual effect-size estimates and shows that the relationship between Necessity beliefs and adherence was significant (*p*<0.05) for 49 (51.0%) of the included studies. Sensitivity analyses revealed that the overall result was not affected when any single finding was omitted.

#### Concerns

There was a significant relationship between Concerns and adherence and fewer Concerns about adverse effects, *OR* = 0.502, 95% CI: [0.450, 0.560], *p*<0.0001. There was significant heterogeneity among the 93 comparisons from 91 studies, *Q*(92) = 481.84, *p*<0.001, suggesting that factors other than chance accounted for a moderate-substantial amount of variance, *I* ^2^ = 80.91%.

**Figure 3:**
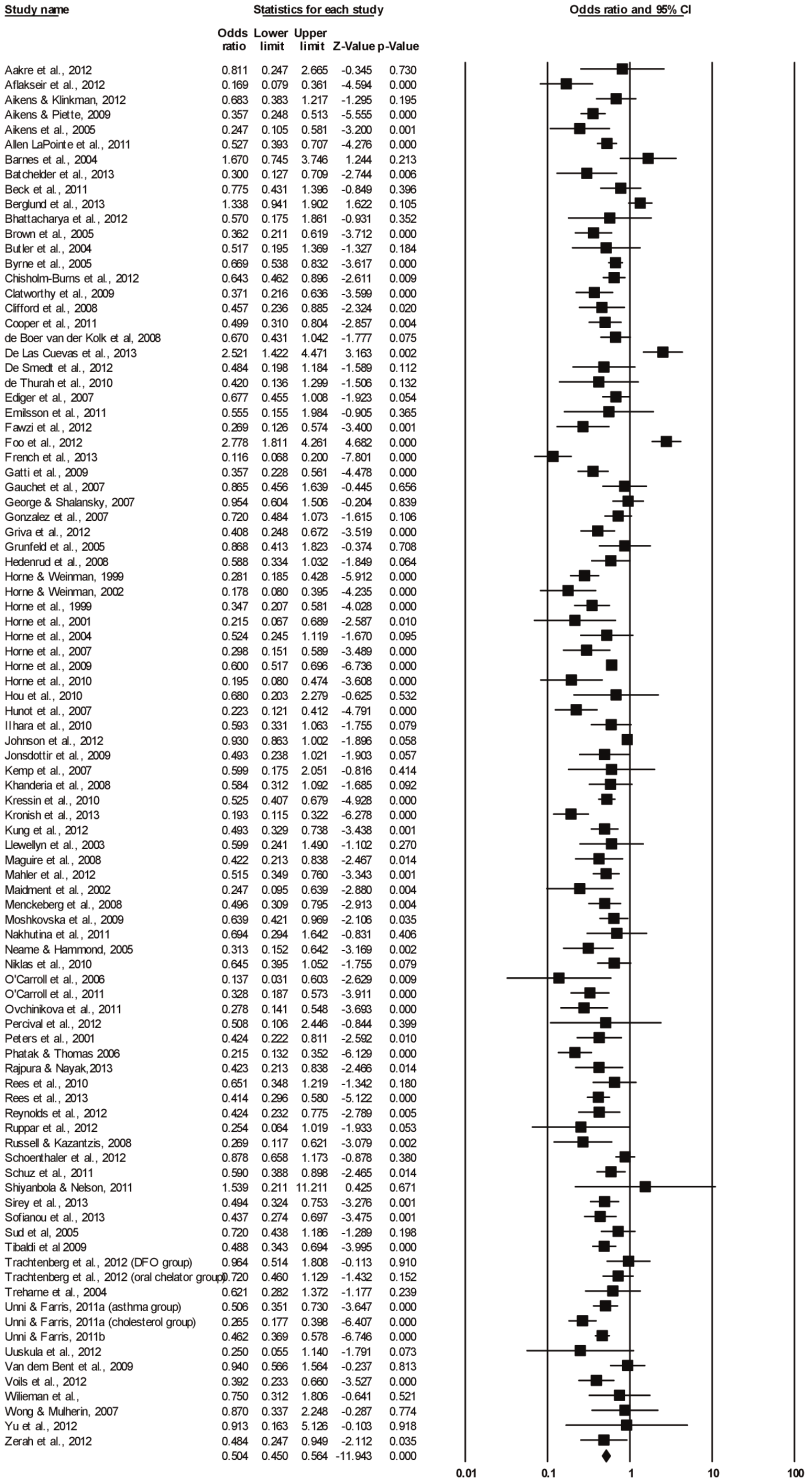
Forest plot of effect sizes for BMQ Concerns and medication adherence.

Figure 3 presents the individual effect-size estimates and shows that the relationship between concerns and adherence was significant (*p*<0.05) for 53 (57.0%) of the included studies. Sensitivity analyses revealed that the overall result did not change when any single finding was omitted.

### Stratification by Long-Term Condition and Measurement

See Tables 2 and 3 for OR stratified by different long-term conditions and adherence measures. Two few studies reported data on the majority of conditions and measures to allow statistical tests for heterogeneity.

**Table 2:** Analyses Stratified By Long-Term Condition.

	*k*	*OR*	(95% CI)	*p*
Necessity				
Asthma	7	2.610	1.802–3.780	<0.001
Bipolar disorder	2	1.624	0.739–3.567	0.227
Blood disorders	3	1.512	0.580–3.944	0.398
Cancer	2	2.313	1.190–4.496	0.013
Depression	8	1.989	1.382–2.862	<0.001
Diabetes	6	1.502	0.930–2.425	0.096
Dialysis/end stage renal disease	3	1.454	0.771–2.742	0.247
Epilepsy	2	0.859	0.284–2.602	0.789
Glaucoma	3	1.697	0.976–2.949	0.061
High cholesterol	2	1.497	0.659–3.401	0.335
HIV	9	1.742	1.242–2.444	0.001
Hypertension	7	1.426	0.980–2.075	0.064
IBD	3	1.775	1.560–2.020	<0.001
Mixed sample	11	1.504	1.249–1.810	<0.001
Organ transplant	5	2.875	1.561–5.294	0.001
Pain	2	1.239	0.468–3.280	0.666
Rheumatoid arthritis	5	3.277	1.106–9.708	0.032
Schizophrenia	2	3.301	1.115–9.777	0.031
Stroke/CHD/acute coronary syndrome	9	1.402	1.022–1.924	0.036
Concerns				
Asthma	6	0.406	0.304–0.541	<0.001
Bipolar disorder	2	0.410	0.250–0.672	<0.001
Blood disorders	3	0.764	0.545–1.073	0.121
Cancer	2	0.771	0.411–1.445	0.417
Depression	8	0.408	0.215–0.772	0.006
Diabetes	6	0.450	0.202–1.003	0.051
Dialysis/end stage renal disease	3	0.509	0.211–1.232	0.134
Epilepsy	2	0.662	0.327–1.339	0.251
Glaucoma	3	0.909	0.258–3.204	0.882
High cholesterol	2	0.598	0.123–2.918	0.525
HIV	9	0.619	0.465–0.824	0.001
Hypertension	6	0.433	0.340–0.552	<0.001
IBD	3	0.612	0.536–0.698	<0.001
Mixed sample	11	0.423	0.339–0.501	<0.001
Organ transplant	4	0.486	0.356–0.503	<0.001
Pain	2	0.620	0.428–0.897	0.011
Rheumatoid arthritis	5	0.608	0.385–0.962	0.033
Schizophrenia	2	0.648	0.410–1.025	0.063
Stroke/CHD/acute coronary syndrome	9	0.518	0.382–0.704	<0.001

*Note.* CHD = coronary heart disease.

**Table 3:** Analyses Stratified by Adherence Measure.

	*k*	*OR*	(95% CI)	*p*
Necessity				
Brief Medication Questionnaire	2	2.350	1.122–4.341	0.022
CQ-R	2	18.327	5.696–58.967	<0.001
Electronic monitoring	3	1.625	0.599–4.412	0.340
MARS	33	1.838	1.581–2.137	<0.001
MASRI	2	2.048	1.390–3.018	<0.001
MMAS	20	1.558	1.305–1.862	<0.001
Pharmacy refill	3	1.668	0.684–4.066	0.260
Concerns				
Brief Medication Questionnaire	2	0.415	0.131–1.321	0.137
CQ-R	2	0.546	0.286–1.044	0.067
Electronic monitoring	3	0.620	0.403–0.946	0.027
MARS	31	0.425	0.362–0.500	<0.001
MASRI	2	0.410	0.251–0.669	<0.001
MMAS	20	0.590	0.426–0.817	0.002
Pharmacy refill	3	0.785	0.630–0.979	0.031

*Note*. CQ-R = Compliance Questionnaire- Rheumatology from de Klerk, van der Heijde, Landewé, van der Tempel, & van der Linden (2003), MARS = Medication Adherence Report Scale Scale from Horne et al., (1999), MASRI = Medication Adherence Self-Report Index from Walsh et al., 2002, MMAS = Morisky Medication Adherence Scale from Morisky, Green, & Levine (1986).

### Methodology/Quality Assessment

See Table 4 for sensitivity analyses.

**Table 4:** Analyses Stratified By Adherence Measure, Study Location, Design and Power.

	*k*	*OR*	(95% CI)	*p*	*I*^2^	Heterogeneity test
Necessity						
UK study	32	2.201	1.786–2.713	<0.001	72.72%***	*Q*(1) = 7.67, *p*<0.05
Non-UK study	64	1.573	1.405–1.761	<0.001	74.79%***	
Concerns						
UK study	31	0.403	0.335–0.485	<0.001	62.75%***	*Q*(1) = 7.61, *p*<0.05
Non-UK study	62	0.555	0.486–0.635	<0.001	82.48%***	
Necessity						
Subjective adherence measure	83	1.737	1.565–1.929	<0.001	75.54%***	*Q*(1) = 0.031, *p* = 0.86
Objective adherence measure	13	1.817	1.114–2.963	0.017	86.20%***	
Concerns						
Subjective adherence measure	81	0.485	0.429–0.549	<0.001	82.84%***	*Q*(1) = 13.55, *p*<0.001
Objective adherence measure	12	0.726	0.609–0.866	<0.001	8.93%	
Necessity						
Prospective/longitudinal	18	1.526	1.243–1.874	<0.001	63.02***	*Q*(1) = 1.82, *p* = 0.18
Cross-sectional	78	1.798	1.595–2.027	<0.001	79.49%***	
Concerns						
Prospective/longitudinal	18	0.449	0.356–0.567	<0.001	70.88%***	*Q*(1) = 1.14, *p* = 0.29
Cross-sectional	75	0.519	0.458–0.588	<0.001	81.28%***	
Necessity						
Low power	18	1.848	1.290–2.646	0.001	46.19%*	*Q*(1) = 0.12, *p* = 0.73
High power	78	1.730	1.550–1.930	<0.001	80.16***	
Concerns						
Low power	17	0.488	0.371–0.643	<0.001	0.00%	*Q*(1) = 0.05, *p* = 0.82
High power	76	0.505	0.448–0.570	<0.001	83.83%***	

Note. *p<.05, ***p<.001 for Q statistic.

#### Study location

Most studies were conducted outside of the UK (*n* = 62; 66.0%). Stronger effects were apparent for both Necessity and Concerns for studies conducted in the UK relative to studies conducted outside of the UK, however the relationship between Necessity and Concerns was significant for both locations. Substantial and significant heterogeneity was present in all analyses.

#### Study design

The majority of studies (*n* = 77, 81.9%) were cross-sectional, with few studies using longitudinal or prospective designs (*n* = 17; 18.1%). Effect sizes were similar for longitudinal/prospective and cross-sectional designs for both Necessity and Concerns. Substanital and signficant heterogeneity was present in all analyses.

#### Measurement of adherence

Eighty-three studies (88.3%) employed measured adherence using self-report, while 11 (11.7%) used other methods. The association between adherence and Concerns was smaller, but still significant, when objective measures were used, and the heterogeneity around this estimate was small. The association between Necessity beliefs and adherence did not differ if objective or subjective adherence measures were used. Heterogeneity around the subjective measures estimates and the objective Necessity estimate was substantial.

#### Statistical power

Eighteen (19.1%) of the studies were classed as having small samples (less than 82). The size of the associations between Necessity and Concerns and adherence were similar for smaller and larger studies. Heterogeneity estimates indicated that variability around the larger samples estimates was substantial. However, the smaller sample estimates were less heterogeneous, with I^2^ values in the small range for Concerns and the moderate range for Necessity beliefs.

### Assessment of Risk of Publication Bias

#### Necessity

**Figure 4:**
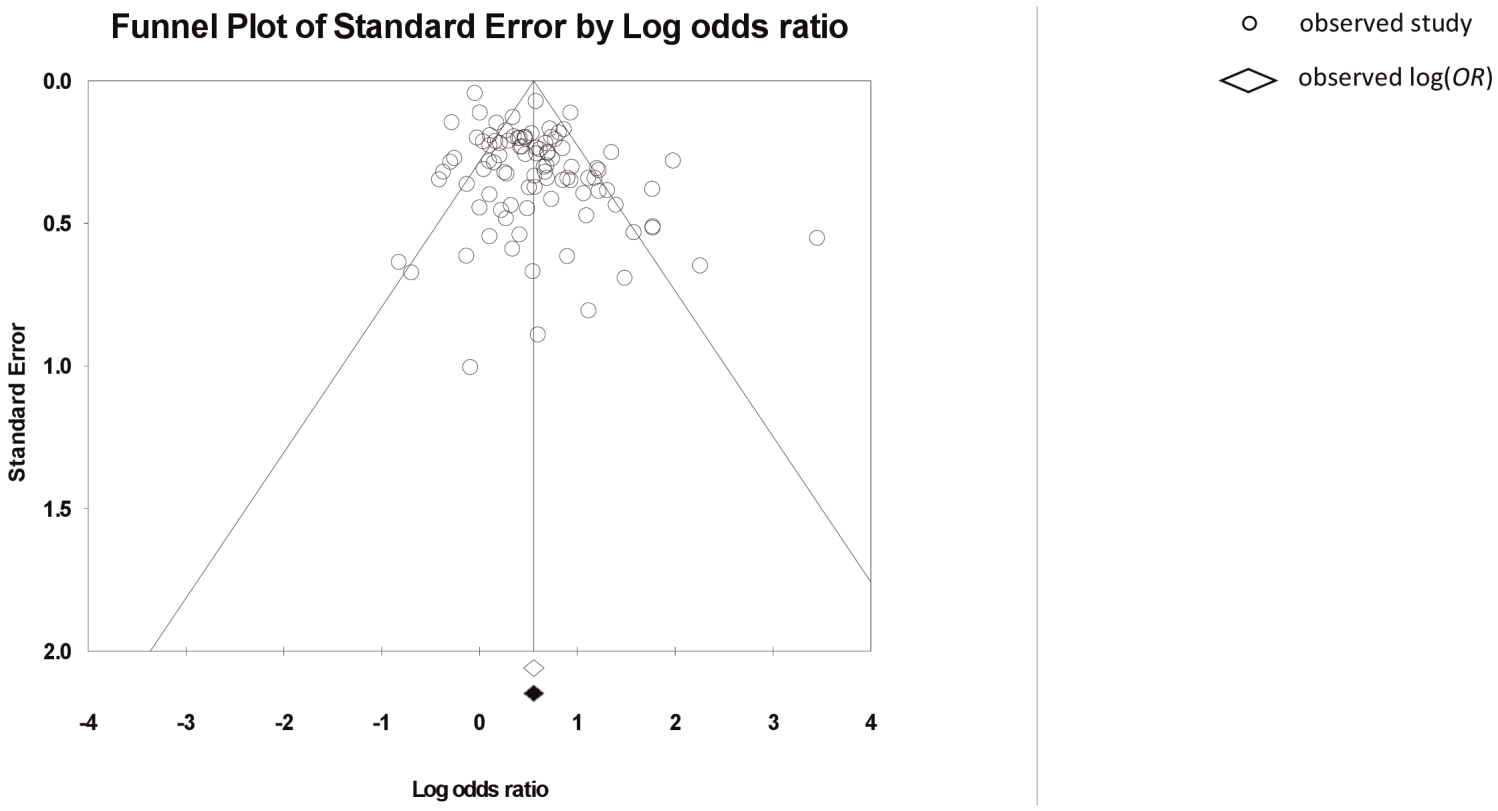
Funnel plot for BMQ Necessity and medication adherence.

The fail-safe *N* (*N_fs_*) was 96, indicating that there would need to be ≥96 unpublished findings of an equal magnitude but opposite direction, to reverse our conclusion that a significant effect exists. Inspection of the funnel plot suggested asymmetry (see Figure 4), however Duval and Tweedie’s trim and fill method did not suggest that studies should be added/removed. Egger’s t-test was significant, *t*(94) = 1.60, *p*<0.001, suggesting the presence of asymmetry.

#### Concerns

**Figure 5:**
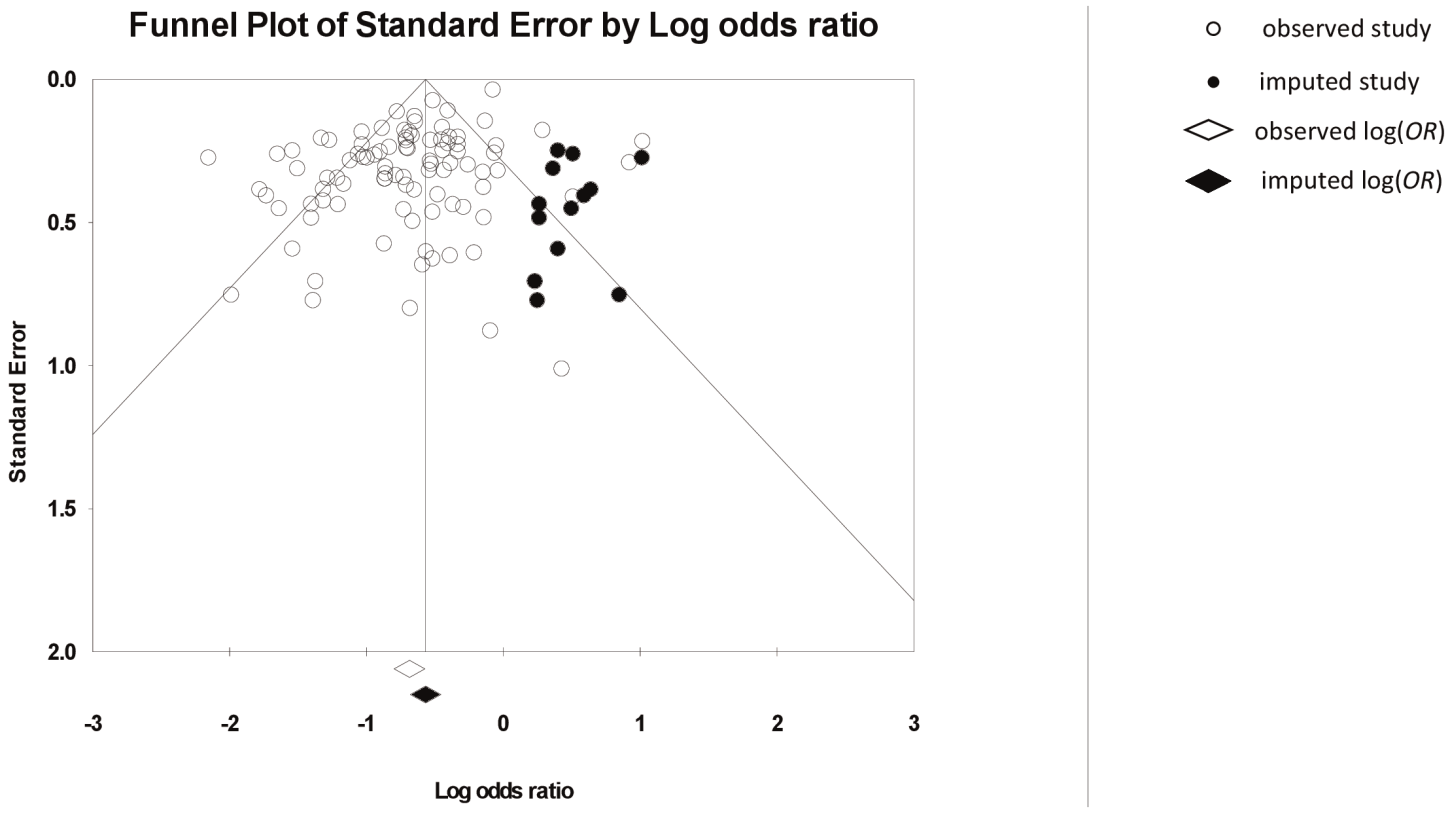
Funnel plot for BMQ Concerns and medication adherence.

The fail-safe *N* (*N_fs_*) was 94, indicating that there would need to be ≥94 unpublished findings of an equal magnitude but opposite direction, to reverse our conclusion that a significant effect exists. Funnel plot inspection suggested the presence of asymmetry (see Figure 5), which was confirmed by a significant Egger’s t-test, *t*(91) = 1.80, *p*<0.001. Further, Duval and Tweedie’s trim and fill method suggested 13 studies should be added/removed to make the funnel plot symmetrical. The location of the imputed studies indicated that the asymmetry may arise from a lack of reporting of studies which find a negative relationship between concerns and adherence. However, the similarity between the adjusted *OR* 0.567 95% CI [0.507, 0.634], which includes the imputed trimmed and filled studies, and the observed *OR* 0.504 95% CI [0.450, 0.564], suggests that any bias does not have a large impact on the findings.

## Discussion

This meta-analytic review indicates that the Necessity-Concerns Framework (NCF) is a potentially useful model for understanding patients’ evaluations of prescribed medicines. The magnitude of the aggregate effect sizes indicates that, for each standard deviation increase in Necessity beliefs, the odds of adherence increases by a factor of 1.7. Conversely, for each standard deviation increase in Concerns, the odds of adherence decreases by a factor of 2.0.

### Strengths and Limitations of the Study

The sensitivity and publication bias analyses conducted confirm our hypothesis that Necessity beliefs and Concerns are associated with adherence/nonadherence to medicines, across a wide range of conditions, medications, and study locations. No research synthesis can transcend the limitations of the primary studies. However, sensitivity analyses confirmed that this association is robust across methodological features; remaining when small, underpowered studies were removed, when only longitudinal/prospective designs were included, and when self-report and non self-report adherence assessments were included separately. The majority of the studies relied solely on self-reported adherence. Self-report measures have high face validity and high specificity for nonadherence, however they may be subject to self-presentation and recall bias [161]. Thus some people may be reporting higher adherence rates than they actually attain. This bias does not diminish our confidence in the finding that beliefs were related to adherence, as there is no evidence that such a bias would be associated with medication beliefs. Indeed some patients with high Concerns and low Necessity beliefs may be expected to incorrectly report high adherence in order to present themselves positively. This pattern would attenuate the relationship found between adherence and medication beliefs, making it less likely that we would find an association between beliefs and adherence. Moreover, given that this relationship remained when non-self report measures were used, we are confident that the observed relationships between beliefs and adherence are not an artifact arising from the limitations of self-report. Only published studies were included, creating a possible bias, since studies submitted for publication may be more likely to have positive results and larger effect sizes. Since for both Necessity beliefs and Concerns, the fail safe N indicated that the number of additional negative findings required to accept our null hypothesis was similar to the number of studies included in this meta-analysis, and there was little suggestion of publication bias through funnel plot analysis, our findings appear to reflect a true relationship between beliefs and adherence.

Stratifying by long-term condition and adherence measurement revealed a need for further studies using objective measures, and highlighted some conditions, for example epilepsy and functional pain syndromes where further research is needed. We do not know whether the Necessity-Concerns Framework will be of equal utility across medications administered by different routes e.g. depot injections, or if practical barriers to care may be of relatively greater importance in some groups using medications administered through different routes.

Eighteen studies assessed whether Concerns and Necessity beliefs could predict adherence using longitudinal/prospective designs. The relationship was not reduced in these studies, supporting the proposal that medication beliefs can influence later adherence as part of the self-regulation of illness [14]. We did not restrict our inclusion criteria to studies published in English. However, our search only identified one study published in any other language, despite the fact that the BMQ was translated into the native language for the study. Cultural values [162] can impact on the way in which individuals interact with the healthcare system. However, variations in treatment necessity and concerns and association between these beliefs and adherence were noted across different countries, languages and cultures. We found that studies outside the UK, where the BMQ and it’s disease-specific modifications have been predominantly developed, found reduced associations between necessity and concerns beliefs and adherence. Further work is needed to investigate potential cultural variations in medication beliefs.

### Implications for Research and Practice

The development of more effective methods for addressing nonadherence is a priority for research and practice [1], [5]. Our findings suggest, that novel interventions to support informed choice and optimal adherence to appropriately prescribed medicines are likely to be more effective if they take account of patients’ beleifs about the treatment and how they judge their personal need for the prescription relative to concerns about ponteial adfverse consequences of taking it. Necessity beliefs and Concerns may trigger intentional nonadherence, for example, if patients decide not to take their medication due to concerns regarding potential or actual adverse consequences, and unintentional nonadherence, (e.g. if patients who believe a medicine is not important for their health forget to take it). Beliefs can have counter-balancing effects on adherence, such as when patients continue to take a medication they believe is essential for their health despite concerns regarding adverse effects ^15^. The challenge now is to develop effective interventions to address patients’ doubts about the necessity for treatment and concerns about adverse consequences in order to enhance adherence. The challenge goes beyond ‘getting patients to take more medicines’. Our findings show that many patients harbour significant, unresolved doubts and concerns about prescribed treatment suggesting a fault-line between patients’ and prescribers’ cultural perceptions of the treatment. Viewed from the perspective of biomedicine, nonadherence may seem irrational. However, from the patients’ perspective, nonadherence may be a ‘common-sense’ response to their implicit appraisal of the treatment. For some patients nonadherence might represent an *informed* choice. In this case the outcome of ‘adherence support’ would be to avoid prescribing an unwanted treatment, to the relief of patient and payer. However, for others, evaluations of treatment necessity and concerns may be based on misconceptions about the illness and treatment.

More detailed studies of patient representations illness and treatment show that, even when treatment evaluations are based on misconceptions they appear to draw on a ‘common-sense’ logic [12], [163], [164]. For example, the need for daily medication may seem less salient when symptoms are absent or cyclical [165]–[167]. Concerns about prescribed medication are not just related to side effects but are common, even when the medication is well tolerated. They are often related to beliefs about the negative effects of medication and include worries about long-term effects, dependence, cost of medication and dislike of having to rely on medicines [14], [167]. Concerns are related to more general beliefs about pharmaceuticals as a class of treatment which are often perceived as intrinsically harmful and over-prescribed by doctors [167], [168]. The package information leaflets, dispensed with many prescription medicines may exacerbate concerns as they list all possible side effects, leaving patients with outstanding questions and making it difficult to understand the likely risk and place them in context with potential benefits [169].

Nonadherence is often a hidden problem. Patients may be reluctant to express doubts or concerns about prescribed medication and to report nonadherence; sometimes because they fear that this will be perceived by the prescriber as a lack of faith in them. The first step to facilitating adherence is therefore to take a ‘no-blame approach’ and encourages an honest and open discussion to identify nonadherence and the reasons for nonadherence [1]. Adherence support should be tailored to the needs of the individual addressing perceptions (e.g. necessity beliefs and concerns) as well as practicalities (e.g. capacity and resources). This can be approached in a three stage process: 1) communicating a common-sense rationale for personal need that takes account of the patient’s perceptions of the illness and symptoms expectations and experiences 2) eliciting and addressing specific concerns and 3) making the treatment as convenient and as easy to use a possible. Interventions attempting to improve adherence by applying these approaches have had encouraging results [142], [170]. Nonadherence remains a fault-line in clinical practice. Consideration of patients’ perceptions of treatment necessity and concerns in prescribing and treatment review is essential to support informed choice and optimal adherence to appropriately prescribed treatment.

## Supporting Information

Supporting Information S1 **PRISMA Checklist.**
(DOC)
